# Levothyroxine Tablet Malabsorption Associated with Gastroparesis Corrected with Gelatin Capsule Formulation

**DOI:** 10.1155/2016/1316724

**Published:** 2016-05-16

**Authors:** David P. Reardon, Peter S. Yoo

**Affiliations:** Department of Pharmacy and Department of Surgery, Yale-New Haven Hospital, New Haven, CT 06510, USA

## Abstract

Treatment of hypothyroidism with levothyroxine sodium often requires multiple dose adjustments and can be complicated by patients with gastric and intestinal dysfunction that limits absorption. In these cases, doses are often titrated higher than commonly used in clinical practice. Multiple formulations of levothyroxine are currently available and some may be preferred in cases of malabsorption. We report a case of a 42-year-old female who presented with a living unrelated kidney transplant evaluation with myxedema while being treated with levothyroxine sodium tablets. She was noted to have gastroparesis secondary to Type I diabetes mellitus which may have contributed to levothyroxine malabsorption. Changing to a gelatin capsule formulation quickly corrected her thyroid function assays. This case suggests that gastroparesis may affect absorption of levothyroxine tablets and the gelatin capsules may be an effective alternative therapy.

## 1. Introduction

Treatment of hypothyroidism with levothyroxine sodium is common in clinical practice and average doses of 100–200 mcg (1.5–2.2 mcg/kg) per day are utilized for replacement or TSH-suppression [1]. Absorption of levothyroxine occurs primarily at the jejunum and ileum through the intestinal mucosa with bioavailability between approximately 62% and 82%. Treatment is primarily done with levothyroxine sodium tablets which have been shown to cause a serum peak within one to three hours after administration [2]. There are many factors that may alter the effectiveness of therapy with malabsorption being a major one [3]. An alternative formulation of levothyroxine sodium is available as an orally administered capsule containing levothyroxine dissolved in glycerin which may offer improved absorption in patients with gastric disorders [4–6]. We report a case of gastroparesis secondary to Type 1 diabetes mellitus leading to significant impairment of levothyroxine sodium tablet absorption and postponement of a living unrelated kidney transplant.

## 2. Case Presentation

A 42-year-old female with a past medical history significant for hypothyroidism secondary to a thyroidectomy, pituitary adenoma, and retinopathy, gastroparesis, and chronic kidney disease, stage V requiring hemodialysis secondary to Type I diabetes mellitus presented for a preoperation appointment for a scheduled living unrelated renal transplantation the following week. Her diagnosis of gastroparesis was made prior to referral to the transplant center but was consistent with her clinical presentation of repeated nausea, vomiting, and episodic weight loss. She was found to be myxedematous with significant edema of the lower extremities, worsening fatigue, a sensation of feeling cold all the time, and a history of dry skin and brittle nails that worsened with the progression of her kidney disease. Her laboratory data was significant for a TSH of 1480 *μ*U/mL (reference range 0.3–4.2 *μ*U/mL), an increase from 180.41 *μ*U/mL three months prior, a free T_4_ of 0.5 ng/dL (reference range 0.8–1.8 ng/dL), and a thyroxine binding capacity of 25.9 *μ*g/dL (reference range 19–28 *μ*g/dL). She was taking levothyroxine 400 mcg (5.9 mcg/kg) and liothyronine 5 mcg, both by mouth once daily separated at least one hour from her other medications and supplements. Her most recent thyroid replacement adjustment was four months earlier by her primary endocrinologist. Her transplant surgery was postponed due to uncontrolled hypothyroidism.

She was referred to a second endocrinologist for further workup and clinical recommendations. It was determined that the patient was highly compliant with her medication regimen and took great care in separating her levothyroxine and liothyronine from other medications and food. As there was no apparent cause of decreased absorption or alterations in metabolism, the decision was made to give a week's worth of weight based levothyroxine sodium tablets with her current formulation and assess a free T4 level prior to administration and 2 hours after with a normal test being denoted by at least a 50% increase in her T4 level. Levothyxine 600 mcg was administered without change in her T4 levels based on previously published data [7]. Next, the tablet formulation of levothyroxine and liothyronine therapy were discontinued and a trial of levothyroxine 300 mcg capsules once daily was started due to the potential for improved absorption prior to initiation of intravenous levothyroxine.

Two weeks after initiation of levothyroxine capsule initiation, the patient's TSH was 42 *μ*U/mL with a free T4 of 1.7 ng/d. Over the next several months, her dose was further adjusted based on TSH and free T4 levels and she ultimately was maintained on a dose of 225 mcg once daily with a TSH of 0.12 *μ*U/mL and a free T4 of 0.8 ng/dL 14 months after changing levothyroxine formulations. She never required intravenous levothyroxine and, six months after initial postponement, she successfully underwent a living unrelated kidney transplant.

## 3. Discussion

There is a significant amount of literature describing the challenges of levothyroxine sodium therapy particularly in the area of malabsorption. Despite this data, there has only been one other documented case report of gastroparesis leading to levothyroxine malabsorption [8]. The patient presented in the case received escalating doses of levothyroxine sodium tablets until he had normalization of his thyroid studies. As other gastric and intestinal causes associated with malabsorption did not seem clinically likely, the authors concluded that gastroparesis should be considered in patients with persistent TSH elevations despite usual levothyroxine doses.

Dose escalation was attempted in the patient presented in this case report without clinical efficacy. After the change in levothyroxine sodium formulation was attempted, the patient had a rapid response in her TSH and T4 levels. She was eventually placed on a maintenance dose of 225 mcg (3.3 mcg/kg) orally once a day. Although this dose is still higher than those commonly used in clinical practice, this was a significant reduction compared to the patient's previous dose.

*Tirosint®* (Akrimax Pharmaceuticals, LLC, Cranford, NJ, USA) is a levothyroxine sodium gelatin capsule that contains T4 dissolved in glycerin. This formulation has been shown to have the most consistent dissolution pattern which results in a more reliable bioavailability. In patients with impaired gastric acid secretion, the gelatin capsule formulation was shown to maintain similar median TSH levels despite a 17% lower dose of levothyroxine compared to a tablet formulation [4].

The role of gastric acid and food interactions with levothyroxine is commonly known with a proposed one-hour delay between administration of levothyroxine and breakfast being recommended [9]. Food and even beverages such as coffee have been shown to retain levothyroxine sodium tablets in the intestinal lumen resulting in a decreased bioavailability [10]. This is likely the reason for the lack of response observed in this patient as subsequent meals may have sequestered appropriately administered doses and decreased absorption.

The gelatin capsule formulation, unlike the tablet formulation, is unaffected by concurrent consumption of coffee [11]. The gelatin capsules may be able to bypass the sequestering mechanism of traditional levothyroxine preparations resulting in better absorption and treatment efficacy in patients with gastric and intestinal malabsorption issues [7]. In this case, the gelatin capsule formulation was effective at treating uncontrolled hypothyroidism despite titration of the tablet formulation.

In patients with uncontrolled hypothyroidism despite adequate treatment titration, gastroparesis should be considered as a possible mechanism for malabsorption, particularly in patients with risk factors such as diabetes mellitus. The gelatin capsule formulation of levothyroxine sodium may be an effective alternative to the tablet formulation in patients with gastroparesis, although larger trials examining this hypothesis are warranted before widespread clinical recommendations can be made.
